# *Konstantinovia* Is Not Monotypic, and a New Attempt to Determine Relationships in Cephaloziellaceae–Scapaniaceae Superclade (Marchantiophyta)

**DOI:** 10.3390/plants13010015

**Published:** 2023-12-19

**Authors:** Vadim A. Bakalin, Vladimir E. Fedosov, Ksenia G. Klimova, Yulia D. Maltseva, Alina V. Fedorova, Seung Se Choi

**Affiliations:** 1 Laboratory of Cryptogamic Biota, Botanical Garden-Institute FEB RAS, Makovskogo Str. 142, 690024 Vladivostok, Russia; fedosov_v@mail.ru (V.E.F.); ksenia.g.klimova@mail.ru (K.G.K.); maltseva.yu.dm@gmail.com (Y.D.M.); 2 Faculty of Biology, Lomonosov Moscow State University, Leninskie Gory Str. 1–12, 119234 Moscow, Russia; 3 Laboratory of Molecular Systematics of Plants, Tsitsin Main Botanical Garden, Russian Academy of Sciences, Botanicheskaya Str., 4, 127276 Moscow, Russia; alina_77777@mail.ru; 4 Team of National Ecosystem Survey, National Institute of Ecology, Seocheon 33657, Republic of Korea

**Keywords:** Lophoziaceae, Obtusifoliaceae, Anastrophyllaceae, molecular phylogeny, Commander Islands

## Abstract

The exploration of liverworts on Bering Island (the westernmost Aleutians) has revealed plants assigned to the recently described and previously monotypic *Konstantinovia*, previously known only from Yunnan Province of China, and belonging to the bigeneric Obtusifoliaceae. The collected plants are described here as *Konstantinovia beringii* sp. nov. The known localities of two species of *Konstantinovia* are separated by more than 6000 km, while the presence of the genus on the Commander Islands is probably a relict. Phylogenetic examination of both collected specimens and new material from other related families resulted in the construction of a fairly well-supported phylogenetic tree for the entire Cephaloziellaceae s.l. + Scapaniaceae s.l. clade. The constructed trees have confirmed the previously stated assumption that it is necessary to segregate one more family within this superclade, described here as Oleolophoziaceae fam. nov.

## 1. Introduction

The enhancement of liverwort phylogenetic data occurs in two ways: by involving new species that were previously completely unexplored and by obtaining sequences of previously unsampled markers in species that have already been used in molecular phylogenetic studies. Utilizing new data allows better resolution and correction of the phylogeny of seemingly well-studied groups. In our case, the unexpected finding of plants resembling the recently described southeastern Sino-Himalayan *Konstantinovia pulchra* Bakalin et Fedosov in the Commander Islands (westernmost section of Aleutians) along with assessment of their systematic position provoked an attempt to further resolve the relationships among already known genera within the superclade Cephaloziellaceae s.l.–Scapaniaceae s.l. The results of these studies are considered in the present paper.

The deep phylogenetic relationships of the traditionally (e.g., [1,2]) treated families Lophoziaceae, Scapaniaceae, and, unexpectedly, Cephaloziellaceae were revealed during the first stages of liverwort phylogenetic studies. In De Roo [3], an analysis of the phylogeny of Lophoziaceae based on a comparison of the plastid *rps*4 gene and the *trn*G intron sequences showed the “embedding” of *Cephaloziella* representatives in the traditionally treated Lophoziaceae. Furthermore, part of the traditionally circumscribed Lophoziaceae was found in a separate clade, subsequently segregated as Anastrophyllaceae [4]. Vilnet et al. [5], in their work on Cephaloziineae, however, concluded that the relationships between Cephaloziellaceae and the Lophoziaceae–Scapaniaceae complex are more distant since the latter turned out to be more closely related to Cephaloziaceae—a point of view not supported in further studies. Considering unstable topologies and weak nodal support, Váňa et al. [6] proposed a compromise in which Scapaniaceae, Lophoziaceae, and Anastrophyllaceae were interpreted in the narrow senses, while all “transitional” elements (like *Obtusifolium*, *Protolophozia*, and *Oleolophozia* with typical lophozioid appearances) were added to Cephaloziellaceae, turning the latter into a ‘superfamily’. A similar view, with the only difference being that the transitional elements were treated as belonging to the Scapaniaceae (making it a superfamily), was adopted by Patzak [7].

After the discovery of a peculiar new genus genetically related to *Obtusifolium* in the mountains of southwestern China, we attempted to revise the taxonomy of families within the superclade Scapaniaceae–Anastrophyllaceae–Lophoziaceae–Cephaloziellaceae, abandoning the concept of superfamily as a grouping incomparable with the other families accepted in liverworts [8]. We assumed that the variation in analyzed markers and an increase in the number of involved markers and specimens could change nodal support values in the resulting phylogenetic trees. However, it was clearly shown that at least two from the pool of conditionally “intermediate” genera should be separated into their own family, Obtusifoliaceae (*Obtusifolium* and *Konstantinovia*). Furthermore, we assumed that in the course of the more detailed study, other genera more closely related to either Cephaloziellaceae or Scapaniaceae would be assigned either to the already known families or to new families [8]. The same was previously predicted by Váňa et al. [6] (p. 1): “Morphologically it is difficult to defend such a diverse family [Cephaloziellaceae] and it should probably be separated into further families”. In particular, this is the case of *Oleolophozia*, the genus that was found in a strongly phylogenetically isolated position by Bakalin et al. [8], but the set of the involved markers and taxa of the traditionally circumscribed Lophoziaceae was considered insufficient for further taxonomic resolutions.

## 2. Results

Nuclear ITS alignment included 358 conservative sites, 621 variable sites, and 464 parsimony-informative sites. The combined *trn*G, *rbc*L, *rps*4, *psb*A, and *trn*L–F alignment of the 69 specimens included 2536 conservative sites, 1555 variable sites, and 1180 parsimony-informative sites.

**Figure 1:**
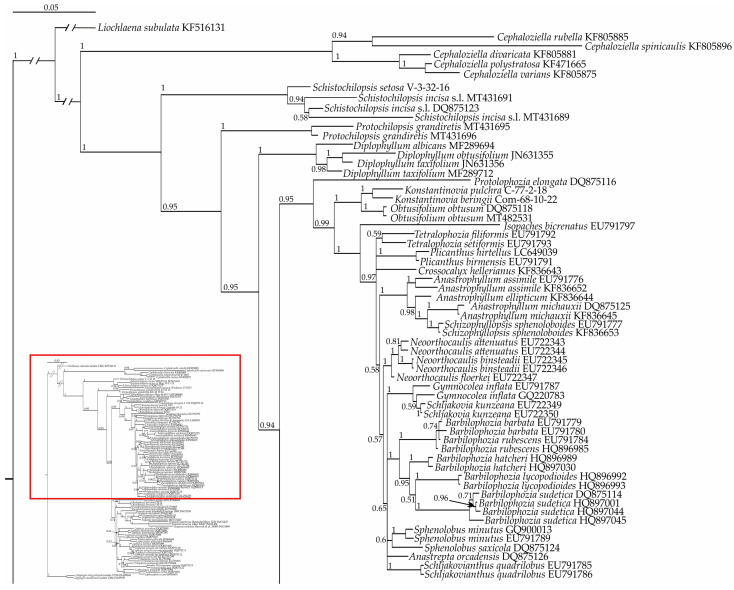

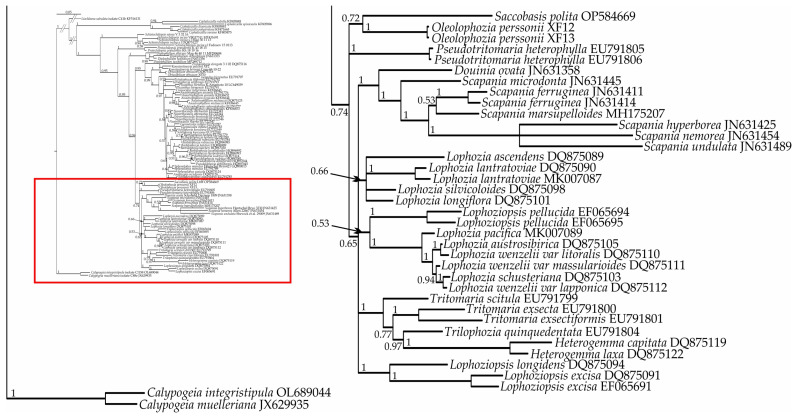
Phylogram obtained from a Bayesian analysis for the listed taxa based on the ITS 1–2 dataset. Posterior probabilities greater than 0.50 are indicated. Taxon names and GenBank accession numbers or vouchers (for the samples studied by the authors) are provided.

**Figure 2:**
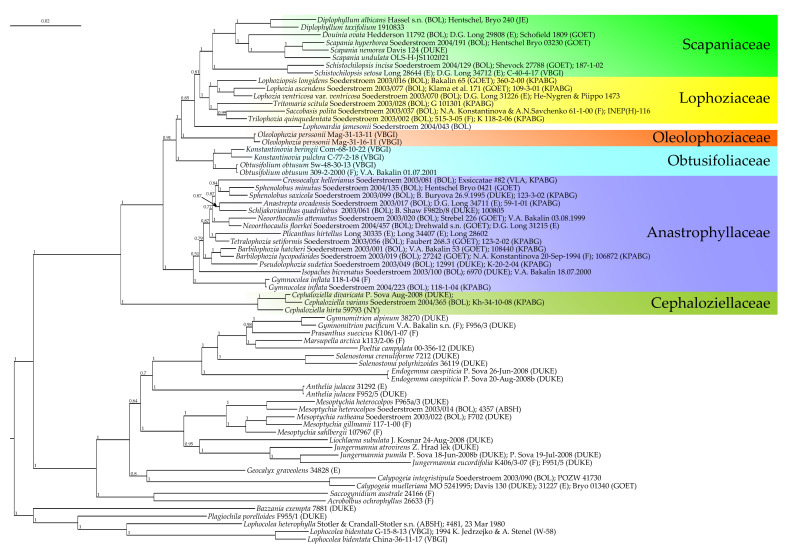
Phylogram obtained from a Bayesian analysis for the listed taxa based on the *trn*G, *trn*L–*trn*F, *rps*4, *psb*A, and *rbc*L dataset. Posterior probabilities greater than 0.50 are indicated. Taxon names and GenBank accession numbers or vouchers (for the samples studied by the authors) are provided.

The general topology of the trees we obtained were in agreement with those presented by Vilnet et al. [9], Shaw et al. [10], Bakalin et al. [11], and Bakalin et al. [8]. In both Bayesian phylogenetic trees (Figure 1 and Figure 2), the target specimen *Konstantinovia* sp. Com-68-10-22 appeared in a maximally supported clade with *K. pulchra* within the maximally supported Obtusifoliaceae clade. The position of the latter within the Anastrophyllaceae + Scapaniaceae clade received moderate support, while the relationships within it were not resolved. The position of the *Oleolophozia* clade composed of *Oleolophozia perssonii* specimens (XF12 and XF13) differed among trees, as inferred from the analyzed datasets. In the ITS-based tree (Figure 1), it was nested in the basal portion of the unsupported clade, corresponding to Lophoziaceae/Scapaniaceae, while in the better resolved and supported tree inferred from the plastid dataset (Figure 2), the *Oleolophozia* clade splits on the next node after the Obtusifoliaceae clade, thus occupying a sister position to the Scapaniaceae + Lophoziaceae clade.

Therefore, the analysis clearly showed that (1) *Oleolophozia perssonii* is sister to the Scapaniaceae + Lophoziaceae clade, and (2) the specimen from Bering Island indeed belongs to the genus *Konstantinovia*, representing a previously undescribed species, which we describe below as *K. beringii*.

## 3. Discussion

### 3.1. On the Circumscription of the Genus Konstantinovia

#### 3.1.1. Morphology

The basic features of *Konstantinovia* are present in *K. beringii*, including: (1) terminal *Frullania*-type branching that looks similar to *K. pulchra* as a strange modification of intercalary branching when one of the branches is distinctly smaller than another and the ventral leaf lobe is only reduced, but not typically absent; and (2) acute leaf lobes with a wide and deep sinus and with a sometimes-present sharp tooth near the antical leaf base. Furthermore, some features differentiate the new species from *Konstantinovia pulchra*, including (1) total absence of ventral branches (probably they are rare and were simply not found); (2) the sharp tooth near the antical base is rare in *K. beringii* and present only in the largest plants and in some leaves; (3) the tooth near the postical leaf base is totally absent (common in *K. pulchra*); (4) underleaves are totally absent in the taxon (or easily deciduous and not found for this reason), versus nearly regularly arranged, although easily deciduous and hidden in rhizoids, subulate to indistinctly bilobed as two closely aligned filaments of different length, sometimes with 1–2 additional basal slime papillae present in *K. pulchra*; and 5) gemmae are virtually absent in *K. beringii*. The morphological comparison of the two *Konstantinovia* species is presented in the Table 1.

All mentioned features might be regarded as extreme ecological modifications of the same species. However, the latter point of view is hardly appropriate (even from the strictly morphological point of view) because plant size greatly overlaps: 1.0–2.2 mm wide in *K. pulchra* versus 0.7–1.5 mm in *K. beringii*. Moreover, the midleaf cell size is even larger in *K. beringii* than in *K. pulchra*: 25–35 × 20–30 µm versus 20–30 × 15–25 µm, respectively. Taking the latter into account along with the genetic differences discussed above and the geographic distance between two populations, we prefer to treat plants from Bering Island as a new species described above. The field observations imply the same: when collecting *K. pulchra* in the Hengduan Mts. in southern China VAB wrote in the envelope in the field “something like *Barbilophozia*”, while initially studying the specimen of *K. beringii* VAB described as unknown *Protolophozia* or “dioicous branches of *Lophoziopsis excisa*?” and misrecognizing the sporadic distribution of antical teeth in leaves as evidence of androecial bracts missing antheridia.

#### 3.1.2. Geographic and Ecological Considerations

The Aleutian Islands, the western part of which is the Commander Archipelago, occupy a special place in the global phytogeographic system [12,13,14,15,16]. On the one hand, this is an alternative to the Asian-American Beringian Land Bridge between floras of two continents, where the Aleutian ‘path’ is much warmer and more humid, in comparison with the cryoxeric conditions in the territory of the Beringian bridge in the Pleistocene–Holocene glacial maxima [13]. On the other hand, it is a chain of disconnected islands that has never been a single land bridge between two continents and a place for the preservation of more southern species in shelters because of insularity. For various groups of plants in the Commander Islands, it is possible to find representatives of either endemic plants or those with locations that are remarkably distant from the main area body [13,17,18].

*Konstantinovia* is the only known genus of liverworts that has a strictly Sino-Himalayan–Aleutian distribution. Even at the species level, this is rare because many taxa occur more widely than Sino-Himalaya + Aleutians due to radiations to Eastern Asia and/or occurrence in western North America. Two liverwort species [19] have similar distribution patterns to that of *Konstantinovia*: *Lejeunea stevensiana* (Yunnan Province of China, Nepal, Bhutan, India, Aleutians, and Ecuador, where the occurrence in the last country may be seriously doubted) and *Plagiochila poeltii* (Xizang, Sichuan and Yunnan Provinces of China, Nepal, India, Aleutians). The generally East Asian taxa that disjunctively occur in the Aleutians and then in Western North America are not rare. With some reservation, that group may be referring to the following taxa (mostly based on the list provided in the section “North Pacific Arc species” in [19] with a few additions): *Acrobolbus ciliatus*, *Douinia plicata* (the occurrence of this taxon in Aleutians is not disjunctive), *Syzygiella nipponica*, *Metasolenostoma orientale*, *Metacalypogeia cordifolia*, *Nardia japonica*, *Plagiochila semidecurrens*, and *Porella fauriei*. In mosses several species also have wide distribution ranges with just a few known localities within it, likely, reflecting their relic (species of the genus *Distichophyllum*, *Pseudoditrichum mirabile* Steere and Z. Iwats., *Oreas martiana* (Hoppe & Hornsch.) Brid., *Schistidium chenii* (S.H.Lin) T.Cao, C.Gao & J.C.Zhao, etc.), several being known from the Sino-Himalaya. However, disjunction between the Sino-Himilayan region and Aleutians do not occur in mosses; thermophylous mosses, reaching the northern limit of their distribution in Aleutians, typically also occur in the Kuril Islands and Kamchatka (*Anomodon longifolius* (Schleich. ex Brid.) Hartm., *Trachycystis flagellaris* (Sull. and Lesq.) Lindb., *Brachymenium nepalense* Hook., *Eurhynchiadelphus eustegia* (Besch.) Ignatov and Huttunen, etc., cf. Fedosov et al. [17]). At the same time, Aleutians houses many mosses with other disjunctive distribution patterns, predominantly Amphioceanic (*Plenogemma phyllantha* (Brid.) Sawicki, Plášek and Ochyra, *Pseudotaxiphyllum elegans* (Brid.) Z. Iwats., *Rhytidiadelphus loreus* (Hedw.) Warnst., and those, having temperate west–western disjunction [20], the latter group hardly reach Commander Islands.

The type localities of the two *Konstantinovia* species are separated by 28 degrees in latitude (26°38′34.7″ N 99°45′16.9″ E in *K. pulchra* and 54°48′46.2″ N 166°38′58.5″ E in *K. beringii*). These two points are situated at distances of more than 6100 km along a geodesic straight line. Such a significant distance, in theory, implies serious differences in climatic conditions. Although taking into account the fact that *K. pulchra* was collected at an altitude of over 3500 m a.s.l. (against 2 m a.s.l. for *K. beringii*) can explain the suggested significant differences. A direct comparison of the selected bioclimates of both sites is given in Table 2. This comparison shows noticeable differences in mean annual temperature, precipitation amount in the driest quarter, and precipitation seasonality, while the majority of the parameters show a surprising similarity in which both species exist. This similarity is clearly seen in the temperature seasonality, the maximum temperatures of the warmest and coldest months, the annual amount of precipitation and the total precipitation of the wettest quarter of a year. In other words, if we assume that members of the genus must have similarity in ecological requirements as an ‘archetype’ (in large genera, this is very commonly not the case), then the ecological requirements of both species turn out to be quite close.

The ecological habitat of *K. beringii* is not unusual in terms of the commonness of habitats on both the Commander Islands [17,18] and the Aleutians as a whole [19]. This is a wet, slightly shaded cliff along the seacoast. However, it should be mentioned that among the relics, the percentage of epilithic taxa increases, and the rocky substrates themselves are often refugia of the preceding floras [21]. In addition, as mentioned above, the Aleutian Islands themselves are also a refugium for many species that occur there at a significant distance from the area cores. Thus, findings such as those described here are quite expected.

In previous papers, we discussed the similar pathways of morphological evolution in different liverwort families in the Sino-Himalaya and in the Holarctic North, expressed in plagiotropic growth and bilobed leaves associated with a greater or lesser reduction in underleaves. While some families are currently sidelined, such as Harpanthaceae [22], Obtusifoliaceae [8], or the herein-described Oleolophoziaceae, other families are widely distributed in the northern Holarctic and Sino-Himalaya (for example, Lophoziaceae, especially its genus *Lophozia*). Moreover, some of these now almost extinct groups could have previously been distributed more widely. Finding *Konstantinovia beringii* in such an isolated locality apart from another species known from Yunnan Province in China suggests that this genus also had a wider distribution and was not a narrow-range endemic of the eastern extremity of the Sino-Himalayas. The fact that this remarkable genus has not been reported from the huge area between Himalayas and Commander Islands, covered by the collection points of the first author, is probably also caused by extinction or a critical decrease in *Konstantinovia* throughout the area of its previous distribution; probably, this genus should be considered as a relict and thus deserves conservation efforts.

#### 3.1.3. On the Phylogenetic Relationships in the Scapaniaceae–Cephaloziellaceae Superclade

In a large clade that includes more-or-less clearly defined Cephaloziellaceae, Scapaniaceae + Lophoziaceae, and Anastrophyllaceae, there are a number of elements that fit, conditionally speaking, between them. In many cases, the nodes containing these genera (e.g., *Gottschelia* Grolle, *Herzogobryum* Grolle, *Lophonardia* R.M.Schust., *Nothogymnomitrion* R.M.Schust., and *Protolophozia* (R.M.Schust.) Schljakov) have low support in the phylogenetic tree, and their position cannot be definitively resolved. Some authors attribute these elements to the Cephaloziellaceae superfamily [6], and some to Scapaniaceae s.l. (essentially equal to the Scapaniaceae superfamily, cf. Patzak et al. [7]). A very different point of view was expressed by us when describing the new family Obtusifoliaceae, which is that if these intermediate elements are assigned to one or the other, the level of morphological heterogeneity and genetic intrafamiliar *p*-distances of one of the ‘superfamilies’ is incomparable with other recognized liverwort families. Therefore, to preserve morphological and genetic consistency, we propose excluding such ‘superfamilies’ and, on the contrary, segregating these distinct elements into narrow families. This is what we did when describing the family Obtusifoliaceae [8]. To date, by significantly increasing the number of involved markers and specimens, we made sure this was the right approach and concluded that it is necessary to describe at least one more family, Oleolophoziaceae, which would include only *Oleolophozia perssonii*. Morphologically, the only representative of this family has one feature different from other species of the ‘Scapaniaceae superfamily’ clade, which is the presence of large, single (1–2), long-persistent oil bodies in the gemmae cells, often with a central eye (thus biconcentric).

Our main phylogenetic tree of Cephaloziellaceae–Scapaniaceaeis (Figure 2) is based on five plastid markers, while a rapidly evolving nuclear ITS region, resulting in a conflicting topology, was not included. The probable reasons of the conflict may be the different inheritance of the nuclear (biparental) and plastid (uniparental) loci and hybridization [23]. In comparison with our previous trees for this group based on two markers each [8], this approach increased the reliability of the presented results but, on the other hand, reduced the number of constituent elements. The *Oleolophozia* clade occupies a position sister to the Scapaniaceae + Lophoziaceae + *Lophonardia* clade, i.e., the basalmost within the Lophoziaceae s.l. clade; the latter is sister to the Obtusifoliaceae clade and their joint grouping is sister to the well supported Anastrophyllaceae clade. Notably, in the present reconstruction, it is inferred from five combined plastid loci, Lophoziaceae is not paraphyletic, as was the one inferred from the two combined markers in our previous study [8]).

The description of *Lophozia perssonii* (=*Oleolophozia perssonii*) was long considered not to be discordant in the genus *Lophozia* s. auct. Although already in the original description [24], a specific feature of the new species was noted—large and few oil bodies in gemmae cells that persist for a long time in the herbarium. Several elder authors [1,2] agreed that *L. perssonii* is a member of the *Lophozia* sect. *Excisae* and morphologically similar to the type species of the section (*Lophozia excisa*, =*Lophoziopsis excisa* in the current understanding). However, this suggestion was not supported by the molecular phylogenetic analysis showing that the species should be placed into a separate genus. The same oil body features plus the bright coloration of the gemmae were repeated in the description of the genus *Oleolophozia* in Söderström et al. [4], while the genus was mostly founded based on molecular data. In addition to the mentioned morphological traits, one ecological feature needs to be mentioned: the species is confined to rocks rich in calcium, a trait that is rare (although present) in *Lophoziopsis*. At the time of genus description, the authors emphasized [4] (p. 51) that “family placement is somewhat unclear but we retain it in Lophoziaceae for now”—a point of view that contradicts the further conclusion by Váňa et al. [6]. The data presented here support the separation of *Oleolophozia* into its own family.

Oleolophoziaceae has a typical ‘lophozioid’ appearance of the habit due to bilobed oblique to almost transversely inserted leaves, reduced (totally absent in *Oleolophozia*) underleaves, ascending to erect growth, obovate, perianth plicate at the mouth. These features are quite common both among Lophoziaceae (*Lophozia*, *Lophoziopsis*, etc.) and Anastrophyllaceae (*Vietnamiella*, *Pseudolophozia*, etc.). Therefore, this type of structure can be considered an evolutionary finding, which ensured the wide distribution of just such a combination of traits in the northern latitudes and mountains south of the Northern Holarctic. Similar morphological adaptations were previously shown in currently ‘suppressed’ Harpanthaceae and Obtusifoliaceae [8,23].

### 3.2. Taxonomy

#### 3.2.1. *Konstantinovia beringii* Bakalin, Fedosov, Klimova et Maltseva sp. nov.

**Figure 3:**
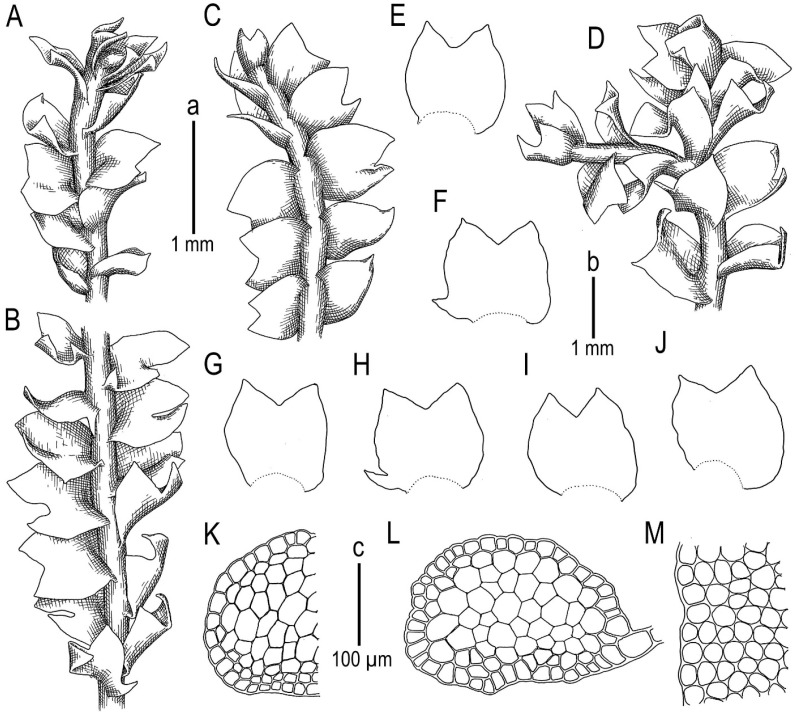
*Konstantinovia beringii* Bakalin, Fedosov, Klimova et Maltseva sp. nov.: (**A**,**D**) upper part of a shoot, fragment, dorsal view; (**B**) middle part of a shoot, fragment, dorsal view; (**C**) upper part of a shoot, fragment, ventral view; (**E**–**J**) leaves; (**K**) stem cross-section, fragment; (**L**) stem cross-section; (**M**) leaf margin cells. Scales: a—1 mm for (**A**–**D**); b—1 mm for (**E**,**F**); c—100 µm for (**K**–**M**). All from Holotype Com-68-10-22 (VBGI).

**Figure 4:**
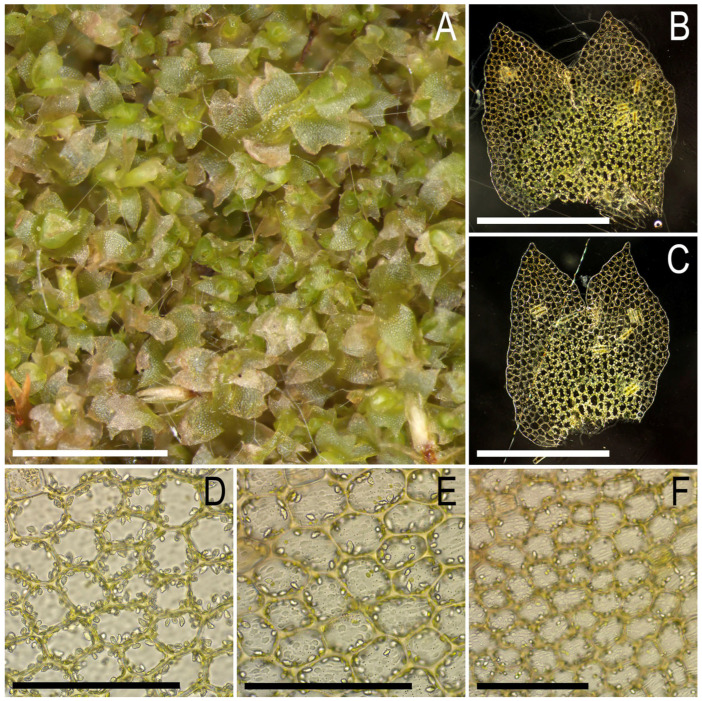
*Konstantinovia beringii* Bakalin, Fedosov, Klimova et Maltseva sp. nov.: (**A**) mat, dorsal view; (**B**,**C**) leaves (photographed with dark field option); (**D**) midleaf cells with oil bodies; (**E**,**F**) papillose cuticle of the midleaf cells. Scales: 2 mm for (**A**); 500 µm for (**B**,**C**); 100 µm for (**D**–**F**). All from Holotype Com-68-10-22 (VBGI).

Description. Plants prostrate to loosely ascending near apices, pale green to yellowish greenish in loose patches, rather soft, (0.7–)1.0–1.5 mm wide and 7–15 mm long, and fragile when dry. Rhizoids sparse to locally abundant, originating evenly from a narrow ventral segment, colorless, not united into fascicles, and obliquely to erect spreading. Stem nearly straight to slightly flexuous, pale greenish, the ventral side not differing in color from the dorsal side, except for sometimes a slight tinge of brownish in the stem’s older parts in the ventral side, branching sparse, terminal of *Frullania* type, instead of ventral leaf lobe (ventral leaf lobe in this case it not totally absent but strongly reduced); stem cross section transversely ellipsoidal, 150–250 × 200–300 μm, with outer cells 15–20 μm in diameter, with the outer wall strongly thickened, other walls thin or only slightly thickened, with large concave trigones, inward cells becoming larger, 5-polygonal (20–)25– 40 μm in diameter, very thin-walled with small and concave to vestigial trigones. Leaves contiguous to somewhat distant, in general obliquely inserted and oriented, insertion line very oblique in ventral side and subtransverse in dorsal; dorsal leaves barely decurrent, leaf lobe apices commonly turned up to the stem apex, whereas the leaf sinus is turned down or loosely folded, thus looking as the most taxa of *Schistochilopsis*; when flattened in the slide transversely ellipsoidal to widely ovate-trapezoidal, well developed 600–700 × 700–800 μm, divided by a widely V- to γ-shaped sinus descending to 1/4–1/3 of the leaf length into two subequal (ventral commonly slightly larger) triangular to loosely gibbous acute lobes, in larger leaves sometimes with an acute and falcate tooth near the antical base, and small plants constantly without additional teeth in the leaf bases. Underleaves absent. Midleaf cells subisodiametric, 25–35 × 20–30 μm, thin-walled with a moderate-in-size triangle to slightly concave or convex trigones, cuticle distinctly and densely papillose; cells along the leaf margin 25–30 μm in diameter, thin-walled, with moderate-in-size concave trigones, outer wall distinctly thickened, cuticle loosely verrucose. Gemmae and generative structures unknown (Figure 3 and Figure 4).

**Figure 5:**
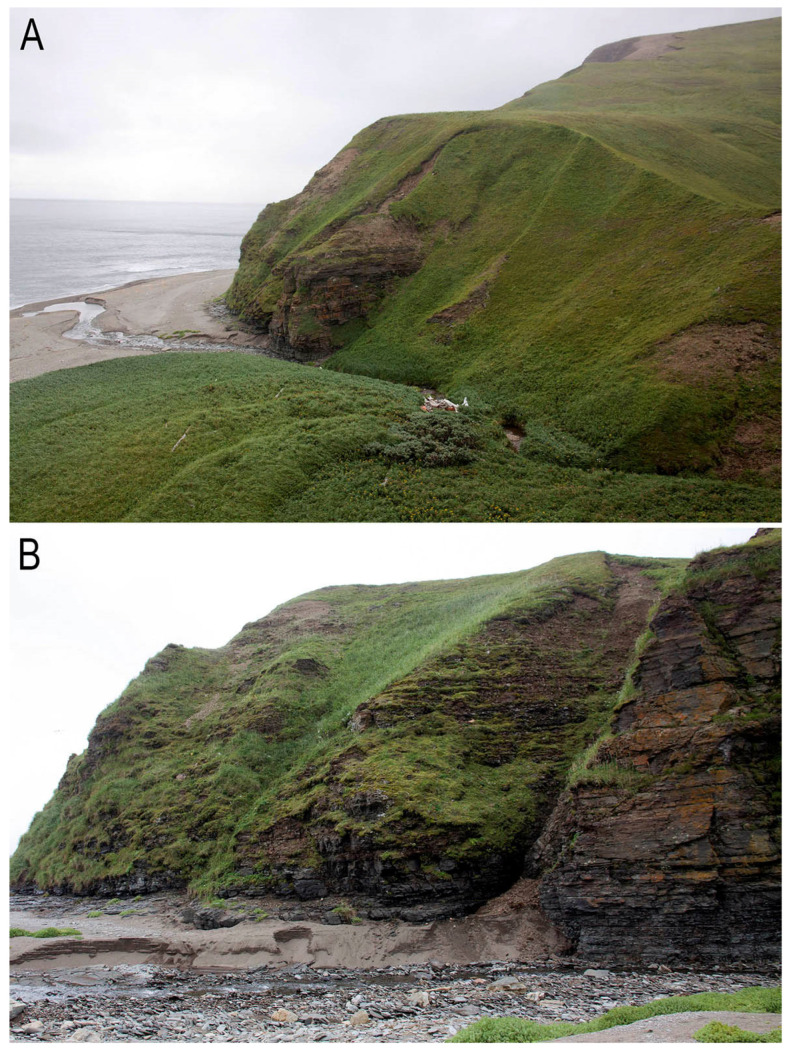
(**A**) Collection place (type locality) of *Konstantinovia beringii* Bakalin, Fedosov, Klimova et Maltseva sp. nov.—river mouth in Mayatnik Bay in the southern part of Bering Island; (**B**) *Konstantinovia beringii* natural habitat: wet to mesic rocky outcrops along river (Photos by A.N. Shienok, 2022).

Holotype: Russia, Kamchatka Territory, Aleutsky District, Commander Islands, Bering Isl., southern part of the island, Mayatnik Bay, river mouth (54°4846.2″ N 166°38′58.5″ E), 2 m alt., wet to mesic rocky outcrops partly covered with coastal vegetation, on sandy soil with plant debris covering rocks, 30 August 2022, K.G. Klimova, Com-68-10-22, (VBGI). Type locality is shown on Figure 5.

Etymology: the species is named in honor of the chief commander Vitus Jonassen Bering (1681–1741)—the discoverer of the Commander Islands who tragically passed away on the island, named after him.

Ecology: Because the species was only collected once, its ecology is not completely known. It was collected on an open rocky outcrop along a small river near its mouth on a border with a seacoast. The proximity of the seacoast causes a high content of sand to be blown onto rocks by the wind in soil, which is a direct substrate for the species.

#### 3.2.2. Oleolophoziaceae Bakalin et Fedosov fam. nov.

Diagnosis. Plants whitish, ascending to erect, soft. Stem and leaves mostly leptodermatic, with thin cell walls and small trigones. Gemmae abundant, orange to orange-brown. Gemmae cells with 1–2 large, long-persisting, sometimes biconcentric oil bodies.

Type genus: *Oleolophozia* L.Söderstr., De Roo et Hedd., Phytotaxa 3: 50, 2010 [4].

Included genera: The family is being created for a single genus, which so far is known to include the single species, *Oleolophozia perssonii* (H.Buch et S.W.Arnell) L.Söderstr., De Roo et Hedd. Therefore, the family is hitherto monotypic.

Type species of *Oleolophozia*: *Oleolophozia perssonii* (H.Buch et S.W.Arnell) L.Söderstr., De Roo et Hedd., Phytotaxa 3: 51, 2010 [4].

Basionym: *Lophozia perssonii* H.Buch et S.W.Arnell, Bot. Not. 97: 382, 1944 (Buch 1944).

## 4. Materials and Methods

The specimen of *Konstantinovia* was collected during field work in the southern part of Bering Island in 2022. The live specimen was transported to Vladivostok to the cryptogamic laboratory of Botanical Garden-Institute FEB RAS. During the identification process, the general appearance of the plant and oil bodies was photographed, and samples were extracted for DNA analysis. For the latter, parts of plants were dried rapidly using silica jelly. Then, the specimen was dried for herbarium storage.

To compare the climate conditions of the type localities of the two *Konstantinovia* species, data on 19 bioclimatic indices were obtained by WorldClim [25,26].

### 4.1. Taxon Sampling

To compile the dataset for molecular phylogenetic analysis, we obtained sequences of six loci (nuclear ITS, plastid *trn*G, *trn*L–F, *rbc*L, *psb*A, and *rps*4) from the target specimen of *Konstantinovia* sp. Com-68-10-22 and added them to the *trn*G–*rps*4 dataset, which was compiled in the course of the study, resulting in the description of the genus *Konstantinovia* [8]. Sequences of the missing loci (*rbc*L, *psb*A) were newly obtained from specimens of *Lophocolea bidentata*, *Lophocolea cuspidata*, *Konstantinovia pulchra*, *Obtusifolium obtusum*, and *Oleolophozia perssonii* included in that dataset, while for GenBank accessions, these sequences were downloaded from GenBank. Accessions from families Lophocoleaceae (*Lophocolea bidentata*, *L. cuspidata*), Lepidoziaceae (*Bazzania exempta*), and Plagiochilaceae (*Plagiochila porelloides*) were selected as the outgroup for plastid tree rooting, following Bakalin et al. [8], while the *Calypogeia* clade (Calypogeiaceae) was used for rooting the ITS tree based on the topology from the same treatment. Specimen voucher details, including GenBank accession numbers, are listed in Table S1.

### 4.2. DNA Isolation, Amplification, and Sequencing

DNA was extracted from dried liverwort tissue using the NucleoSpin Plant II Kit (Macherey-Nagel, Germany). Amplification was performed using an Encyclo Plus PCR kit (Evrogen, Moscow, Russia) with the primers listed in Table 3.

The polymerase chain reaction was performed in a total volume of 20 µL, including 1 µL of template DNA, 0.4 µL of Encyclo polymerase, 5 µL of Encyclo buffer, 0.4 µL of dNTP mixture (included in the Encyclo Plus PCR Kit), 13.4 µL (for *trn*L–F, *trn*G, *psb*A and *rps*4)/12.4 µL (for ITS 1–2 and *rbc*L) of double-distilled water (Evrogen, Moscow, Russia), 1 µL of dimethylsulfoxide/DMSO (for ITS 1–2 and *rbc*L), and 0.4 µL of each primer (forward and reverse, at a concentration of 5 pmol/µL). Polymerase chain reactions were carried out using the protocols listed in Table 4.

Amplified fragments were visualized on 1% agarose TAE gels by EthBr staining and purified using the Cleanup Mini Kit (Evrogen, Moscow, Russia). The DNA was sequenced using the ABI PRISM^®^ BigDye™ Terminator Cycle Sequencing Ready Reaction Kit (Applied Biosystems, Waltham, MA, USA) with further analysis of the reaction products following the standard protocol on an automatic sequencer 3730 DNA Analyzer. (Applied Biosystems, Waltham, MA, USA) in the Genome Center (Engelhardt Institute of Molecular Biology, Russian Academy of Sciences, Moscow).

### 4.3. Phylogenetic Analyses

Datasets were aligned using MAFFT ver. 7.490 [34] with standard settings and then edited manually in BioEdit ver. 7.2.5 [35]. All positions of the final alignment were included in the phylogenetic analyses. Absent data at the ends of regions and missing loci were coded as missing data. Phylogenetic trees were reconstructed using Bayesian inference (BA) with MrBayes ver. 3.2.7 [36]. Topologies of phylogenetic trees inferred from the plastid loci *trn*G (680 bp), *rps*4 (609 bp), *psb*A (1147 bp), *rbc*L (1343 bp), and *trn*L–F (346 bp), datasets were congruent and did not show any supported conflict, so we combined them in the concatenated dataset (69 accessions, 4126 positions of the alignment), while the tree for nuclear ITS (101 accessions, 1037 positions of the alignment) was built separately due to different localizations and sets of included accessions. Indel data were scored for individual partitions using the simple indel coding approach [37] in SeqState 1.4.1. [38].

Bayesian analyses were performed by running two parallel analyses, and models were sampled throughout the GTR model space (nst = mixed). Within the combined plastid dataset, separate partitions were set for noncoding (*trn*G intron, *trn*L–F) and coding (*rps*4, *rbc*L, and *psb*A) loci, and indels were considered a separate partition in both datasets. The analysis consisted of four Markov chains and was run for 2.5 million generations, and trees were sampled every 500th generation. The first 2500 trees in each run were discarded as burn-in. The convergence between runs was previously assessed as an average split deviation frequency value lower than 0.01, reached before 1 million generations. Additionally, ESS values were checked using Tracer v.1.7.2 [39] to be higher than 200. The obtained trees were visualized and configured in FigTree v1.4.4 [40].

## 5. Conclusions

Although establishing Oleolophoziaceae advances the taxonomy of the Scapaniaceae-Cephaloziaceae superclade, several lineages at the tentative familial level are still awaiting comprehensive taxonomic decisions. In particular, the phylogenetic position of *Lophonardia jamesonii* (=*Lophozia jamesonii*) (Figure 2) remains unclear. In our reconstruction, its phylogenetic position was estimated based on sequences of two markers downloaded from GenBank. Perhaps, this species (or genus, other representatives of which have not been studied phylogenetically) should also be referred to a separate family, but thus far we have refrained from such a decision, and since only two markers out of four are included in the phylogenetic study, and these data originated from a single specimen, we have no way to confirm its identification to. Moreover, *L. jamesonii* is not the type species for the genus *Lophonardia* (its type is *Lophonardia caespitosa* R.M. Schust. = *Lophonardia laxifolia* (Mont.) L. Söderstr. et Váňa), so both taxa should be studied to be certain about the name to be applied to the new family.

Another unclear component of the Scapaniaceae–Cephaloziellaceae superclade is the position of *Protolophozia elongata*, which clearly belongs to the ‘Scapaniaceae superfamily’ and is closely related neither to Cephaloziellaceae nor to any other of the formally recognized at the familial level lineage. This taxon may also be in its own family not yet described. However, as we have even poorer sequence data for this taxon, for which only ITS1, 2 are available in GenBank, we doubt that this marker is suitable for establishing phylogeny at the familial level. Therefore, familial placement of the genus *Protolophozia* remains to be studied.

These two examples show how far we are still from a satisfactory understanding of the phylogeny of the Scapaniaceae–Cephaloziellaceae superclade. Further involvement of new material and new markers for species already studied by molecular methods will undoubtedly improve resolution and support of deeper nodes within the Scapaniaceae–Cephaloziellaceae superclade and will probably lead to the description of more than one new family. Bechteler et al. [41] recently published the bryophyte time-tree based on 228 nuclear genes, where the topology of the suborder treated in the present account is based on fewer species and does not agree with our main tree based on plastid marker study in some traits. This example shows the imperfectness of both attempts, and work in this direction should therefore be continued.

## Figures and Tables

**Table 1:** The morphological comparison of two *Konstantinovia* species.

Trait	*Konstantinovia beringii*	*Konstantinovia pulchra*
Ventral branches	Not present (at least unknown)	Sparse
Tooth near antical leaf base	Rare	Common
Tooth near postical leaf base	Absent	Sparse
Underleaves in sterile shoots	Absent	Present, nearly regular
Gemmae	Absent (unknown)	Sporadically present

**Table 2:** The comparison of bioclimatic variables (as described in M&M section of the paper) in the *loci classici* of the two *Konstantinovia* species.

Bioclimates	Data for *Konstantinovia pulchra* Type Locality	Data for *Konstantinovia beringii* Type Locality
BIO1 = Annual Mean Temperature, °C	5.0625	1.76667
BIO4 = Temperature Seasonality (standard deviation × 100)	548.76358	617.13169
BIO5 = Max Temperature of Warmest Month, °C	17.6	13.4
BIO6 = Min Temperature of Coldest Month, °C	−7.4	−8.6
BIO12 = Annual Precipitation, mm/year	859.00	916.00
BIO15 = Precipitation Seasonality (Coefficient of variation)	64.11567	26.12135
BIO16 = Precipitation of Wettest Quarter, mm/quarter	396.00	305.00
BIO17 = Precipitation of Driest Quarter, mm/quarter	51.00	166.00

**Table 3:** Primers used in polymerase chain reaction (PCR) and cycle sequencing.

Locus	Sequence (5′-3′)	Direction	Reference
ITS 1–2 nrDNA	CGGTTCGCCGCCGGTGACG	forward	[27]
ITS 1–2 nrDNA	GATATGCTTAAACTCAGCGG	reverse	[28]
*trn*G cpDNA	ACCCGCATCGTTAGCTTG	forward	[29]
*trn*G cpDNA	GCGGGTATAGTTTAGTGG	reverse	[29]
*rps*4 cpDNA	TACCGAGGGTTCGAATCCCT	forward	this study
*rps*4 cpDNA	ATGTCCCGTTATCGAGGACCT	reverse	[30]
*rbc*L cpDNA	ATGTCACCACAAACGGA	forward	[31]
*rbc*L cpDNA	GTATCTATTGTTTCATATTC	reverse	this study
*psb*A cpDNA	GACGAGTTCCGGGTTCGA	forward	[32]
*psb*A cpDNA	TGGAATGGGTGCATAAGG	reverse	[32]
*trn*L–F cpDNA	CGAAATTGGTAGACGCTGCG	forward	[33]
*trn*L–F cpDNA	TGCCAGAAACCAGATTTGAAC	reverse	[33]

**Table 4:** The protocols of PCR-reactions.

Initial denaturation	3 min–94 °C	
Denaturation	30 s–95 °C	35 cycles
Annealing	20 s (*trn*L–F), 30 s (*trn*G, ITS 1–2), 1 min (*rbc*L, *rps*4, *psb*A)	
	at 50 °C (*rbc*L), 56 °C (*trn*G, *psb*A), 58 °C (*trn*L–F), 60 °C (ITS 1–2), 62 °C (*rps*4)	
Elongation	30 s–72 °C (ITS 1–2, *trn*L–F, *trn*G), 1 min (*rbc*L, *rps*4, *psb*A)	
Final elongation	3 min–72 °C	

## Data Availability

Data are contained within the article and Supplementary Materials.

## Supplementary Materials

The following supporting information can be downloaded at: https://www.mdpi.com/article/10.3390/plants13010015/s1, Table S1: The list of taxa, specimen vouchers and GenBank accession numbers.
